# Oral health status, related behaviours and perceived stress in xerostomia, Sicca and Sjögren’s syndromes patients - a cross-sectional study

**DOI:** 10.1186/s12903-024-04224-7

**Published:** 2024-04-15

**Authors:** Indre Stankeviciene, Lina Stangvaltaite-Mouhat, Jolanta Aleksejuniene, Diana Mieliauskaite, Ieva Talijuniene, Irena Butrimiene, Ruta Bendinskaite, Alina Puriene

**Affiliations:** 1 Institute of Dentistry, Faculty of Medicine, Vilnius University, Vilnius, Lithuania; 2 Oral Health Centre of Expertise in Eastern Norway, Oslo, Norway; 3 Department of Oral Health Sciences, Faculty of Dentistry, The University of British Columbia, Vancouver, Canada; 4 Department of Personalised Medicine, State Research Institute Centre for Innovative Medicine, Vilnius, Lithuania; 5 Clinic of Rheumatology, Orthopaedics Traumatology and Reconstructive Surgery, Institute of Clinical Medicine, Faculty of Medicine, Vilnius University, Vilnius, Lithuania

**Keywords:** Adults, Dry mouth, Mouth dryness, Xerostomia, Sjögren’s syndrome, Oral health

## Abstract

**Background:**

Mouth dryness increases the risk of some oral health-related conditions. Furthermore, it is unclear if patients with dry mouth engage in appropriate oral health-related behaviours. The study examined oral health, related behaviours, and perceived stress in dry-mouth patients and compared them to matched controls without mouth dryness.

**Methods:**

Information about 182 dry-mouth patients and 302 age- and sex-matched subjects was retrieved. Three dry mouth groups: xerostomia, Sicca syndrome and Sjögren’s syndrome, were formed based on patient self-reported and objectively assessed symptoms. The World Health Organization’s Oral Health for Adults and Perceived Stress Scale (PSS-10) questionnaires inquired about sociodemographic characteristics, oral health-related behaviours, and self-perceived stress. Clinical oral health assessments included: caries experience measured as total numbers of decayed (DS), missing (MS), filled surfaces (FS), number of remaining teeth, erosive tooth wear and extent of periodontal pocketing. Data were analyzed using bivariate and multivariable tests.

**Results:**

The dry-mouth participants had higher mean (SD) DMFS scores than their matched controls: xerostomia patients vs. controls: 74.6 (34.4) and 66.3 (35.4), Sicca syndrome patients vs. controls: 88.3 (34.0) and 70.1 (33.9), and Sjögren’s syndrome patients vs. controls: 95.7 (31.5) and 74 (33.2). In comparison to controls, individuals with Sicca and patients with Sjögren’s syndromes had lower mean (SD) number of remaining teeth, 15.9 (10.1) vs. 21.7 (8.4) and 13.8 (10.0) vs. 20.1 (9.2), and a lower mean (SD) extent of periodontal pocketing, 20.7 (28.6) vs. 41.1 (31.0), and 21.2 (24.1) vs. 34.8 (34.2), respectively. Xerostomia, Sicca syndrome and Sjögren’s syndrome patients had higher odds of using fluoridated toothpaste; OR 1.8 (95%CI 1.1–2.9), OR 5.6 (95%CI 1.7–18.3) and OR 6.9 (95%CI 2.2–21.3), respectively. Participants with Sjögren’s syndrome had lower odds of the last dental visit being within the last year; OR 0.2 (95%CI 0.1–0.8).

**Conclusions:**

Dry-mouth patients had higher caries experience and fewer teeth than comparison groups but a lower extent of periodontal pocketing. Even though more participants with dry mouth used fluoridated toothpastes, their oral health-related behaviours were not optimal.

## Introduction

As societies age worldwide, dry mouth conditions become a point of interest in research [1–3]. Besides older age, dry mouth was also associated with systemic diseases, use of medications, radiotherapy in the neck or head region, and unhealthy lifestyle, including smoking and high stress, therefore, dry mouth patients are considered a vulnerable population for oral diseases [2, 4–7]. Although the term ‘dry mouth‘ is often used to refer to all dry mouth-related conditions, a distinction needs to be made between specific dry mouth types. Xerostomia is diagnosed in individuals who self-report symptoms of dry mouth [7]. Sicca and Sjögren’s syndromes are diagnostic terms often used interchangeably, although these conditions are different [2]. Sicca syndrome is diagnosed when objective signs of oral and ocular dryness are present [2]. According to our previous study, 25% of individuals with Sicca syndrome also had Sjögren’s syndrome [8]. Sjögren’s syndrome is an autoimmune disease that impacts the exocrine glands, with 95% of patients experiencing mouth dryness and/or dry eyes [2, 5].

Higher dental caries rates and lower numbers of retained teeth are frequently observed in dry mouth patients [9–11]. Also, there are some indications that those with impaired salivation have a greater risk for dental erosion [10, 12]. The relationship between mouth dryness and periodontal disease still remains unclear, as some studies reported a positive relationship, while others did not find such an association [13–16]. Furthermore, individuals may be more at risk of developing various systemic diseases due to their physical, psychological, and social environments, such as poor living conditions and taking health risk behaviours [17]. The development of oral diseases follows a similar pattern. Establishing good oral health-related behaviours is of importance in preventing oral diseases [18]; yet only a few studies have investigated behavioural patterns in patients with dry mouth conditions. A study focused on patients with Sjögren’s syndrome reported that despite oral health behaviours, individuals with Sjögren’s syndrome had poorer oral health than control subjects. However, this study included only 20 patients and 20 control participants [19].

Moreover, stress has been identified as a common risk factor for oral and general diseases. Stress may impact health through a direct pathway which relates to unhealthy behaviours usually practiced to cope with stress, such as having unhealthy diet, smoking, and abusing alcohol. Such risk behaviours dysregulate the body’s homeostasis, for example, causing hyposalivation and xerostomia [20, 21]. In addition, stressful life events may be a risk factor for developing Sjögren’s syndrome [22]. It is still unknown whether dry-mouth conditions have an impact on the most common oral diseases, whether individuals with dry mouth engage in appropriate oral health-related behaviours, and perceive adequate stress levels. The current study examined oral health, related behaviours, and perceived stress in individuals with xerostomia, Sicca syndrome, and Sjögren’s syndrome and compared them to sex and age-matched subjects without mouth dryness. We hypothesized that participants with dry-mouth conditions have higher stress levels, poorer oral health and its related behaviours.

## Methods

### Study participants

Data were extracted from two cross-sectional datasets; 2017–2019 Lithuanian National Oral Health Survey (LNOHS) and 2020–2022 dry mouth study. The dry mouth study selected adult subjects from five capital hospitals: a specialised dental care hospital, a rheumatological centre, and three hospitals providing long-term care for patients with chronic, non-acute medical conditions. The study inclusion criteria were self-reporting mouth dryness assessed by a question: How often do you have dry mouth? (responses: ‘often’ or ‘always’), and consenting to participate in the study.

Considering the mean differences in caries experience between dry mouth groups, the minimum sample size calculation employed the G-power calculator considering 95% power and α = 5%. This calculation showed that we needed to recruit a minimum of 126 participants. In the dry mouth study, we managed to recruit 127 cases, and additional 55 cases were selected from the Lithuanian National Oral Health Survey (LNOHS), which employed a stratified random sampling from five biggest Lithuanian cities and ten randomly selected periurban/rural areas, one in each of the Lithuanian counties (details published elsewhere) [8, 23].

In the LNOHS, subjects with mouth dryness were identified in the same way as in the dry mouth study. To support analyses, three dry mouth groups were created. The xerostomia group comprised individuals who experienced dry mouth ‘often’ or ‘always’ and did not have Sicca or Sjögren’s syndromes. The Sicca syndrome group included participants with hyposalivation indicated by ≤ 0.1 ml/min of saliva production over 15-minute period following the unstimulated whole sialometry test and Schirmer’s test over 5-minute period showing ≤ 5 mm in at least one eye, and excluding participants with a Sjögren’s syndrome diagnosis. The Sjögren’s syndrome group consisted of subjects with a confirmed diagnosis according to the 2016 ACR/EULAR criteria [24]. We found that a portion of participants with Sjögren’s syndrome also experienced Sicca symptoms, and decided to include these patients in the Sjögren’s syndrome group; otherwise, some patients with Sjögren’s syndrome would have needed to be excluded, compromising internal validity. The detailed procedures of unstimulated whole sialometry, Schirmer’s test, and diagnosing Sjögren’s syndrome were presented elsewhere [8]. The comparison groups included controls which were age and sex-matched, and the control subjects for each of the dry mouth groups were selected from the LNOHS dataset [8]. In both studies, all data were collected by one trained and calibrated examiner (IS). The intra-examiner reliability was assessed using the intra-class correlation coefficient (ICC) calculated by comparing duplicate recordings of 10 randomly selected patients, which were not included in the main study, with 2 weeks between repeated clinical examinations. The ICC values indicating intra-examiner agreement were as follows: for the total numbers of decayed surfaces (ICC = 1.00,) missing surfaces (ICC = 0.99,) filled surfaces (ICC = 1.00,) teeth with 4 + mm periodontal pockets (ICC = 0.95 and for the teeth with signs of erosive tooth wear (ICC = 0.95) [23, 25].

### Self-reported data

The World Health Organization’s Oral Health Questionnaire for Adults [26] was used to inquire about participants’ sociodemographic characteristics (sex, age, education in years) and several oral health-related behaviours. These behaviours were measured as follows: frequency of tooth brushing (once a day or less ‘0’, twice a day or more ‘1’); use of interdental floss or toothbrush (no ‘0’, yes ‘1’); use of the fluoride toothpaste (no ‘0’, yes ‘1’); last dental visit within last year (no ‘0’, yes ‘1’); frequency of sweet consumption (a few times a day ‘5’ and every day ‘4’ were referred to as the ‘high’ consumption of sweets in univariate analysis, those who ate sweets several times a week ‘3’ or once a week ‘2’ comprised the ‘moderate’ category, and those consuming several times a month ‘1’ or rarely/never ‘0’ were defined as a ‘low’ sweet consumption. In the binary logistic regression, the original coding was used.

The stress levels were measured by the Perceived Stress Scale (PSS-10), which underwent forward-backward translation from English to Lithuanian languages [27] and subsequent validation. The construct validity of the Lithuanian version of the Perceived Stress Scale was evaluated employing the Exploratory Factor Analysis (EFA) and setting the Eigen value > 1.0 as the threshold for the factor (dimension) extraction. According to the Lee 2012 review of the psychometric adequacy of the perceived stress scale, we expected either one or, alternatively, a 2-factor solution [28]. In addition, to get a better insight into the scale’s dimensions, we employed the Varimax rotation and acquired dimension-based item loadings. Our EFA produced a two-factor solution, each representing clear dimensions (here factors). The ‘Helplessness’ dimension included six items, and the ‘Self-efficacy’ dimension included four items, where the first dimension explained 38.1% and the second dimension explained 20.5% of the scale’s variance. The ‘Helplessness’ dimension included the following items (loadings): item 1 (loading = 0.744), item 2 (loading = 0.816), item 3 (loading = 0.783), item 6 (loading = 0.681), item 9 (loading = 0.681) and item 10 (loading = 0.828.) The ‘Self-efficacy’s dimension items (loadings) were as follows: item 4 (loading = 0.779), item 5 (loading = 0.752), item 7 (loading = 0.727) and item 8 (loading = 0.606.) Our scale’s validation-related findings were in accordance with several earlier studies performed in different countries and diverse samples, which most commonly reported a two-factor PSS-10 structure. Our two-factor/dimension solution explained 59% of the total variance in stress levels and this finding is also in accordance with previous studies. To sum up, our validation-related testing indicates that the Lithuanian PSS-10 scale version is a valid measure.

### Clinical oral examinations

For the clinical assessment of oral conditions, a plane mouth mirror and a CPITN periodontal probe were used as advised by WHO. Dental status was evaluated at a surface level as total numbers of decayed (DS), missing (MS) and filled surfaces (FS). Subsequently, the total DMFS summed all components reflecting one’s overall caries experience [26]. In addition, the number of teeth with signs and levels of erosive tooth wear were also recorded following the WHO methodology [26]. The percentage of teeth with periodontal pockets of 4 + mm indicated the extent of periodontal pocketing.

### Statistical analyses

Analyses were performed using IBM SPSS version 28.0 software (IBM, NY, US), while age- and sex-matched control participants for comparison groups were selected using R 4.1.0 (R Core Team, 2021) package. The mean and standard deviation (SD), as well as the median and inter-quartile ranges (IQR) were calculated for continuous variables. The Chi-square test was used for categorical, and independent sample t-test or Mann Whitney U test were used for continuous outcome variables. The patient groups and their controls were compared in regard to sociodemographic characteristics, oral health-related behaviours and self-perceived stress. The Mann-Whitney U test compared oral health status between patients with dry mouth-related conditions and their controls selected independently for each dry mouth condition. For each dry mouth condition, multivariable binary logistic regression models tested oral health-related behaviours as independent predictors while adjusting their effects for the socio-demographic characteristics. For the multivariable binary logistic regression models, we selected the oral health-related potential predictors based on the literature, and all behavioural predictors in spite of their bivariate significance were included in the multivariable models. The adjusted effects of predictors are presented as Odds Ratios (OR) with their 95% confidence intervals (CI).

## Results

The final dataset included 182 cases and 302 age- and sex-matched participants in the comparison groups. Of all dry-mouth cases, 24% (*n* = 114) were allocated to the xerostomia group, 5% (*n* = 24) to the Sicca syndrome group, and 6% (*n* = 31) to the Sjögren’s syndrome group. A total of 18 (58%) of participants in the Sjögren’s syndrome group also had Sicca syndrome.

Table 1 summarises results of socio-demographic characteristics, oral health-related behaviours, and perceived stress among dry mouth groups and their matched controls. Half of the participants with xerostomia, 67% with Sicca syndrome, and 39% with Sjögren’s syndrome brushed their teeth twice a day; 55%, 42%, and 42%, respectively used interdental care measures. A higher proportion of participants with xerostomia than controls (56% vs. 42%), Sicca syndrome (73% vs. 35%), and Sjögren’s syndrome (80% vs. 44%) used fluoridated toothpaste. In addition, a higher proportion of participants from the Sjögren’s syndrome group than in the comparison group reported that their last dental visit was more than 12 months ago (52% vs. 24%).

**Table 1:** Comparison of socio-demographic characteristics, oral health-related behaviours, and stress levels in xerostomia, Sicca syndrome, Sjögren’s syndrome patients and their controls

Determinants		Xerostomia vs. controls N (%)		*p*-values	Sicca syndrome vs. controls N (%)		*p*-values	Sjögrens’s syndrome vs. controls N (%)		*p*-values
**Socio-demographic characteristics**										
Sex	Total	114 (100)	228 (100)	0.931	24 (100)	48 (100)	1.000	31 (100)	62 (100)	1.000
	Females	84 (74)	169 (74)		18 (75)	36 (75)		27 (87)	54 (87)	
	Males	30 (26)	59 (26)		6 (25)	12 (25)		4 (13)	8 (13)	
Age	Total	114 (100)	228 (100)	0.957	24 (100)	48 (100)	0.838	31 (100)	62 (100)	0.089
	Mean (SD)	61.2 (12.0)	60.8 (11.0)		61.8 (13.7)	61.3 (9.3)		69.1 (19.4)	65.2 (12.1)	
	Median (interquartile range)	63 (15)	63 (15)		64 (13)	64 (11)		72 (24)	71 (14)	
Education	Total Mean (SD)	114 (100)	228 (100)	0.845	24 (100)	48 (100)		31 (100)	62 (100)	0.960
		14.1 (3.0)	14.1 (3.1)		13.7 (2.5)	14.1 (2.6)	0.600	14.2 (4.1)	14.3 (3.6)	
	Median (interquartile range)	14 (4)	14 (4)		14 (5)	14 (4)		15 (4)	15 (4)	
**Oral health-related behaviours**										
Tooth brushing frequency	Total Twice a day or more	114 (100)	228 (100)	0.702	24 (100)	48 (100)	0.067	31 (100)	62 (100)	0.303
		57 (50)	119 (52)		16 (67)	21 (44)		12 (39)	31 (50)	
	Once a day or less	57 (50)	109 (48)		8 (33)	27 (56)		19 (61)	31 (50)	
Using dental floss or interdental brushes	Total	114 (100)	228 (100)	0.878	24 (100)	48 (100)	0.616	31 (100)	62 (100)	0.882
	yes	63 (55)	124 (54)		10 (42)	23 (48)		13 (42)	27 (44)	
	no	51 (45)	104 (46)		14 (58)	25 (52)		18 (58)	35 (56)	
Using fluoridated toothpaste	Total	114 (100)	228 (100)	0.014**	24 (100)	48 (100)	0.004**	30 (100)	62 (100)	0.001**
	Yes	64 (56)	96 (42)		16 (73)	17 (35)		24 (80)	27 (44)	
	No	50 (44)	132 (58)		6 (27)	31 (65)		6 (20)	35 (56)	
Frequency of consumption of sweets	Total	114 (100)	228 (100)	0.804	24 (100)	48 (100)	0.338	31 (100)	62 (100)	0.219
	Low	74 (65)	156 (68)		15 (65)	19 (53)		9 (29)	18 (29)	
	Moderate	32 (28)	57 (25)		7 (30)	11 (30)		15 (48)	20 (32)	
	High	8 (7)	15 (7)		1 (5)	6 (17)		7 (23)	24 (39)	
Last dental visit	Total 12 months ago or earlier	114 (100)	228 (100)	0.263	24 (100)	48 (100)	0.729	31 (100)	62 (100)	0.008**
		69 (60)	152 (67)		16 (67)	30 (62)		15 (48)	47 (76)	
	More than 12 months ago	45 (40)	76 (33)		8 (33)	18 (38)		16 (52)	15 (24)	
**Stress levels (measure**: PSS-10 – Perceived stress scale)										
**PSS-10 scores**	Total Mean (SD) Median (interquartile range)	114 (100)	228 (100)		37 (100)	74 (100)		31 ( 100)	62 (100)	
		17.0 (6.9) 17 (8)	16.3 (6.7) 16 (8)	0.446	16.5 (7.0) 16.0 (9)	16.9 (5.5) 17.5 (8)	0.625	19.6 (7.8) 20 (9)	17.2 (5.9) 18 (7)	0.101

*Mann-Whitney U test; **Chi-square test

Table 2 compares oral health-related conditions between dry mouth groups and their matched controls. In comparison to controls, a higher mean DMFS was in participants with xerostomia, Sicca syndrome, and Sjögren’s syndrome, the latter had higher mean DS and MS, but lower FS mean values. In addition, individuals with Sicca syndrome and Sjögren’s syndrome had a lower mean number of remaining teeth than their matched controls. The mean extent of periodontal pocketing of participants with Sicca syndrome and Sjögren’s syndrome were lower than the corresponding means of their controls.

**Table 2:** Oral health status- comparisons among xerostomia, Sicca syndrome, Sjögren’s syndrome patients and their controls

Dental status		Xerostomia vs. comparison group N (%)		*p* values	Sicca syndrome vs. comparison group N (%)		*p* values	Sjögrens’s syndrome vs. comparison group N (%)		*p* values
DMFS	Total	114 (100)	228 (100)	0.037*	24 (100)	48 (100)	0.032*	31 (100)	62 (100)	0.003*
	Mean (SD)	74.6 (34.4)	66.3 (35.4)		88.3 (34.0)	70.1 (33.9)		95.7 (31.5)	74.0 (33.2)	
	Median (interquartile range)	73 (54)	60 (58)		86 (62)	73 (54)		105 (56)	75 (54)	
DS	Mean (SD)	2.7 (6.3)	3.2 (7.8)	0.370	3.9 (5.5)	2.7 (4.6)	0.251	5.5 (11.4)	1.4 (2.9)	0.015
	Median (interquartile range)	0 (2)	1 (3)		1.5 (5)	1 (4)		1 (5)	0 (1)	
MS	Mean (SD)	40.7 (40.6)	35.1 (40.0)	0.233	43.8 (38.0)	39.7 (32.7)	0.344	65.2 (42.7)	27.1 (18.4)	0.001
	Median (interquartile range)	35 (52)	30 (54)		46 (45)	35 (44)		61 (77)	27 (25)	
FS	Mean (SD)	31.2 (21.3)	28.0 (20.1)	0.207	37.0 (33.0)	26.4 (18.6)	0.903	22.6 (23.5)	45.4 (36.9)	0.002
	Median (interquartile range)	29 (31)	26 (28)		35 (43)	26 (31)		13 (42)	41 (52)	
Number of teeth	Total	114 (100)	228 (100)	0.421	24 (100)	48 (100)	0.018*	31 (100)	62 (100)	0.005*
	Mean (SD)	20.6 (9.1)	21.4 (8.8)		15.9 (10.1)	21.7 (8.4)		13.8 (10.0)	20.1 (9.2)	
	Median (interquartile range)	23 (11)	24 (10)		19 (19)	24 (11)		13 (17)	21 (12)	
Extent of teeth with signs of erosive tooth wear	Total	114 (100)	228 (100)	0.412	20 (100)	47 (100)	0.856	25 (100)	58 (100)	0.984
	Mean (SD)	36.2 (35.0)	39.5 (36.3)		43.8 (38.2)	45.3 (28.5)		37.7 (33.5)	37.3 (32.1)	
	Median (interquartile range)	40.5 (69)	39.5 (71)		39 (79)	48 (39)		39 (59)	38 (63)	
Extent of periodontal pocketing	Total	107 (100)	218 (100)	0.637	20 (100)	47 (100)	0.002*	25 (100)	58 (100)	0.040*
	Mean (SD) Median (interquartile range)									
		34.7 (30.5)	33.6 (30.8)		20.7 (28.6)	41.1 (31.0)		21.2 (24.1)	34.8 (34.2)	
		28 (45)	25 (48)		9 (33)	38 (52)		18 (31)	29 (51)	

Mann-Whitney U test; DMFS: total numbers of decayed, missing and filled surfaces; DS: decayed surfaces; MS: missing surfaces and FS: filled surfaces

Table 3 presents findings from binary logistic regression analyses examining associations between several oral health-related behaviours and dry mouth conditions when adjusted for socio-demographic characteristics. Participants with xerostomia had almost two times higher odds, and participants with Sicca syndrome had almost eight times higher odds of using fluoridated toothpaste than their controls. In addition, participants with Sicca syndrome were seven times more likely to brush their teeth twice a day or more frequently. The participants with Sjögren’s syndrome had seven times higher odds of using fluoridated toothpaste, but 80% lower odds of a last dental visit within the last 12 months than control participants.

**Table 3:** Binary logistic regression analyses: associations between oral health-related predictors and the presence of xerostomia, Sicca syndrome, and Sjögren’s syndrome (predictor effects adjusted for socio-demographic characteristics)

Determinants	Xerostomia Model summary: Nagelkerke R2 = 0.05	Sicca Syndrome Model summary: Nagelkerke R2 = 0.36	Sjögren’s Syndrome Model summary: Nagelkerke R2 = 0.32
	Adjusted ORs (95% CI)	Adjusted ORs (95% CI)	Adjusted ORs (95% CI)
**Socio-demographic characteristics**			
Sex (females vs. males)	1.1 (0.6; 2.0)	5.0 (0.8; 33.2)	1.5 (0.2; 10.5)
Age (years)	1.0 (0.9; 1.0)	1.0 (0.9; 1.1)	1.0 (1.0; 1.1)
Education (years)	1.0 (0.9; 1.1)	1.0 (0.7; 1.2)	1.0 (0.8; 1.1)
**Oral health-related behaviours**			
Tooth brushing frequency (twice + daily vs. less frequently)	0.8 (0.5; 1.3)	7.1 (1.1; 45.1)	0.7 (0.2; 2.3)
Using dental floss or interdental brushes	1.2 (0.7; 2.2)	0.3 (0.1; 1.5)	2.7 (0.7; 10.2)
Using fluoridated toothpaste (‘yes’ vs. ‘no’)	1.7 (1.0; 2.8)	7.6 (1.7; 34.3)	7.4 (2.0; 26.6)
Last dental visit in last year (‘no’ vs. ‘yes’.)	0.6 (0.4; 1.1)	0.6 (0.1; 3.3)	0.2 (0.1; 1.7)
Frequency of sweets consumption (continuous)	1.0 (0.9; 1.2)	0.9 (0.5; 1.7)	1.3 (0.9; 1.9)

## Discussion

The current cross-sectional study examined oral health status, oral health-related behaviours, and self-perceived stress in participants with xerostomia, Sicca syndrome, and Sjögren’s syndrome and compared dry-mouth patient groups with age- and sex-matched controls. We observed that participants having any of the three dry mouth-related conditions had poorer oral health as indicated by their higher caries experience and more missing teeth. In contrast, those with Sicca and Sjögren’s syndromes had better periodontal health than their matched controls. Of importance, oral health-related behaviours were not optimal in the majority of dry mouth patients. Although Sjögren’s syndrome patients were more likely to use fluoridated toothpaste, they tended having less regular dental visits. Our findings only partially support our hypotheses as oral health status in dry-mouth participants was worse than in controls in regards to three out of the four oral conditions and two out of five oral health-related behaviours. However, no significant differences were found among patient and control groups regarding perceived stress levels.

Some of our study’s limitations were potential selection bias due to a cross-sectional study design and potential information bias due to self-reporting. In addition, the cross-sectional study design did not allow us to make any causal inferences. As commonly chosen in cross-sectional studies, we calculated ORs, given these are probability ratios, the true risks of our dry mouth patients might be overestimated. Another limitation might be related to our xerostomia group by including into it individuals who complained of having dry mouth ‘often’ and ‘always’ (common recommendation). However, if we had chosen a different cut-off point for inclusion, for example, also including into this group less severe cases, our findings might have been different.

In addition, we did not have accurate information (potential information bias) about the duration our participants experienced symptoms of mouth dryness, as many participants could not remember this with any accuracy. Similarly, another potential information bias might relate to the cases of Sjögren’s syndrome, because these patients also could not determine the onset of their disease. Furthermore, some of our participants with Sicca symptoms also had the Sjögren’s syndrome. This occurrence is not surprising, as hyposalivation and ocular dryness are common symptoms in Sjögren’s syndrome patients. Another limitation is that we included information from two datasets where slightly different sampling methods were used: the LNOHS study used stratified random sampling, while the dry mouth study recruited a convenience sample from five preselected hospitals. On the positive side, the same questionnaire and the same clinical type of examinations were used in both studies and all clinical examinations were performed by the same standardized examiner.

Higher caries experience rates were observed in participants with dry mouth conditions than in control participants. Our findings are in line to a Danish study which reported a median DMFS value of 83 for those with Sjögren’s syndrome and 43 for the control group [19], and in the Xin et al. Chinese study, mean DMFS values for Sjögren’s and non-Sjögren’s groups were 47 and 33, respectively [29]. In our study, the median DMFS values were higher; in participants with Sjögren’s syndrome, it was 96, while in the comparison group it was 74. In comparison to the two above-mentioned studies, our participants were older, which can partially explain our higher median DMFS value. In our study, those with Sjögren’s syndrome and their controls had mean ages of 69 vs. 65 years, while in a Danish study, the means were 60 and 56, and in the Chinese study, corresponding mean values were 51 and 50 years. One of the possible reasons why caries experience is higher in dry mouth participants than in controls may be changes in the microbiota due to dry mouth conditions. A 2023 study suggested that such changes are specific for patients with Sjögren’s syndrome, while another study mentioned that dysbiosis is seen in all dry mouth patients despite the etiology of their conditions [30, 31].

Currently, oral health-related information about Sicca syndrome patients is scarce. This may be due to the earlier mentioned fact that Sicca syndrome is often considered synonymous to Sjögren’s syndrome, also it is possible that the latter condition is prioritized in research due to its relationship with autoimmunity. A study examining dental caries in diabetes mellitus patients found that xerostomia was significantly associated with higher caries experience [32]. However, there is a lack of studies directly comparing caries between xerostomia and non-xerostomia individuals. It is important to mention that many earlier studies examined xerostomia’s potential impact on dental status where mouth dryness was measured objectively (hyposalivation) instead of using patients’ self-reports (self-perceived dryness).

Our patients with Sicca and Sjögren’s syndromes had significantly lower numbers of present teeth than their matches, and this finding is in accordance with a Dutch study reporting significantly more of edentulous patients with Sjögren’s syndrome than in the control group. Also, the mean number of extracted teeth during a 13-year period was higher in the Sjögren’s syndrome group than in the control group [33]. The authors concluded that this was due to reduced salivary flow, consequently leading to the development of dental caries and eventually to a tooth loss. In our study, Sjögren’s syndrome was associated with lower odds of timely dental visit, consequently lack of regular dental professional care might have led to higher extraction rates.

In our study, we found significantly lower odds for extensive periodontal pocketing in the Sicca and Sjögren’s syndrome groups versus the participants from the comparison groups. A systemic review with meta-analysis revealed that although those with Sjögren’s syndrome had higher plaque rates and gingivitis, no significant mean differences in periodontal pocketing and clinical attachment loss were found between Sjögren’s syndrome patients and their controls [34]. One possible explanation might be the co-occurrence of periodontal diseases and dental caries at the same teeth which consequently might lead to extraction [35]. In our study, such potential co-occurrence could have resulted in higher overall caries experience including lower number of remaining teeth and lower rates of periodontal diseases, as the latter measure related to the numbers of remaining teeth.

The need to assess the quality of oral hygiene in patients with mouth dryness was emphasized by other studies [34]. Even though dry-mouth patients have increased risk of some oral health diseases, in general, their oral health-related behaviours were not optimal. Only 39–67% of the participants with dry mouth brushed their teeth twice a day, 42–55% of them used interdental measures, and 48–67% did not have a dental visit within the last year. We also found that those with dry mouth were two to eight times more likely to use fluoridated toothpaste than participants from the comparison groups; most likely dentists encouraged their high-risk dry mouth patients to practice regular use of fluoridated toothpaste twice daily, however the compliance with such recommendation we observed only in Sicca syndrome patients. We found that participants with Sjögren’s syndrome had lower odds of visiting a dentist on a regular basis. In contrast, a 2001 study comparing oral health-related behaviours between Sjögren’s syndrome patients and controls found that patients had more frequent dental visits, and a higher proportion of them brushed teeth more than twice a day [36]. However, this study was performed in a Scandinavian country where compliance with oral health-related behaviours might be superior. Important to consider that for those with dry mouth to practice good oral health behaviours might be more challenging, as in addition to having mouth dryness, some of them may experience chronic fatigue and pain [37]. These challenges might lead to decreased motivation to perform physically demanding regular oral self-care. In spite of being in a high-risk patients group, as indicated by having higher rates of dental caries and a lower number of teeth, our subjects with dry mouth conditions tended to have inadequate oral health-related behaviours.

Our findings suggest a further need to examine in more detail other potential determinants of high dental caries and tooth loss in dry mouth patients. We believe that clinicians should emphasize to their dry mouth patients the importance of prevention of oral diseases. Furthermore, national population-based oral health prevention programs should be established with a focus on including high-risk dry mouth patients.

## Conclusions

Dry-mouth patients had higher overall caries experience, fewer remaining teeth, and a lower extent of periodontal pocketing than participants from comparison groups. Despite a higher usage of fluoridated toothpaste among patients with dry mouth than in the comparison groups, their oral health-related behaviors were suboptimal, indicating a need for improvement.

## Data Availability

The data supporting this study’s findings are available upon reasonable request to the corresponding author (IS, indre.stankeviciene@mf.vu.lt).
